# Severe but Self-Limiting Polyarthralgia with Functional Impairment Following ChAdOx1 nCov-19 Vaccination in an Elderly Recipient

**DOI:** 10.3390/vaccines9111220

**Published:** 2021-10-21

**Authors:** Joel Ern Zher Chan, Anand Irimpen

**Affiliations:** 1 Investigator Clinic, Port Lincoln, SA 5606, Australia; jirimpen@investigatorclinic.com.au; 2 Flinders and Upper North Local Health Network, Port Augusta, SA 5700, Australia; 3 Adelaide Medical School, Adelaide, SA 5000, Australia

**Keywords:** COVID-19, SARS-CoV-2, vaccine, Oxford/AstraZeneca, ChAdOx1 nCov-19, elderly patient, reactogenicity, adverse reactions, polyarthralgia, polymyalgia rheumatica

## Abstract

A 79-year-old female patient with no pre-existing rheumatological conditions presented with severe functional impairment secondary to polyarthralgia, most likely an adverse reaction following her first dose of Oxford/AstraZeneca ChAdOx1 nCov-19 vaccination against SARS-CoV-2, the causative agent of Coronavirus Disease 2019 (COVID-19). The presentation mimicked clinical features of polymyalgia rheumatica and was distinctive in its pattern and delayed onset. Its severity in an elderly patient was significant against trial findings of decreasing reactogenicity of ChAdOx1 nCov-19 vaccine with increasing age, and traumatic to the patient. Acute phase reactants were elevated, consistent with recent similar reports among mostly elderly, female patients. New onset rheumatological conditions and flares of pre-existing, well-controlled conditions had been well established in COVID-19 and, to a lesser extent, post-vaccination. Viral arthralgias as a distinct clinical entity in COVID-19 is only beginning to be recognized. It could be that this case report represents a similar entity which occurs following vaccination against SARS-CoV-2. Despite this, the benefits of vaccination continue to outweigh such risks, although this case report is important for providing understanding of clinical progression when such reactions occur, aiding in patient discussions and clinical decisions to weigh up further investigations or empirical treatment against reassurance and close monitoring.

## 1. Introduction

The Oxford/AstraZeneca chimpanzee adenovirus-vectored ChAdOx1 nCov-19 vaccine appears to confer similar immunogenicity among younger and older adults, but is better tolerated in older adults, with local and systemic adverse reactions being less commonly reported among older adults (aged ≥56 years) than among younger adults (aged 18–55 years) [1]. Reactogenicity appears to be decreased following the second dose compared to after the first dose [1].

In evaluating the reactogenicity of the ChAdOx1 nCov-19 vaccine, Ramasamy et al. enrolled 560 healthy adults in three different age groups as part of the initial phase 2/3 trial, including 49 healthy adults in the ≥70 age group who received two standard doses of ChAdOx1 nCov-19 vaccine [1]. Solicited adverse reactions were recorded by enrolled trial participants for seven days post-vaccination and 28 days for unsolicited reactions. Fatigue, joint pain, and muscle ache were among the solicited adverse reactions; diarrhea, a known adverse reaction to adenovirus-vectored vaccines [2], was not a solicited adverse reaction. Among 49 participants in the ≥70 age group (Table S7), 20 participants reported fatigue after the first dose, 7 reported joint pain, and 9 reported muscle aches, respectively. Among these, three participants reported that their fatigue resulted in mild to moderate limitation. No other participants reported limitation in their activity as a result of these adverse reactions, including among the seven participants who reported joint pain. No serious adverse events related to the vaccine occurred as part of the trial, as of 26 October 2020.

In the initial phases of the vaccination rollout in Australia, people aged 60 years and above without contraindications were only eligible for the Oxford/AstraZeneca ChAdOx1 nCov-19 vaccine, due to the potential increased risk of thrombosis with thrombocytopaenia (previously vaccine-induced immune thrombotic thrombocytopaenia), following ChAdOx1 nCov-19 vaccination among people under 60 years old [3].

Here, we present a case of an elderly patient with no prior rheumatological diagnosis who presented with polyarthralgia and severe functional impairment following her first dose of ChAdOx1 nCov-19 vaccine.

## 2. Case Presentation

The patient is a 79-year-old Caucasian female who, at her baseline, goes for regular, long walks with her husband, without aids or assistance. She had no known exposures to SARS-CoV-2 and resides in a rural city 650km away from Adelaide, the capital city of South Australia. There had been enhanced community testing capacity and uptake for SARS-CoV-2 testing across South Australia, with no cases of COVID-19 detected in the community since December 2020; all cases were among returned international travelers within designated quarantine facilities in Adelaide [4,5,6].

On 30 April 2021, the patient received her first dose of ChAdOx1 nCov-19 vaccine in a vaccination clinic in her residential city. She developed mild fatigue within 12 h of receiving the vaccine, with no functional impairment, but which persisted through to her presentation to our primary care clinic on 18 May 2021—18 days after her first dose of ChAdOx1 nCov-19 vaccine. She had developed worsening polyarthralgia from 16 May 2021, affecting her neck, shoulders, knees, and right hip, requiring wheelchair use on the day of presentation. She denied muscle aches. There was concurrent diarrhea at the onset of the polyarthralgia, which was beginning to resolve by the time of presentation; there was no fever, abdominal pain, blood in stools, nausea and vomiting, or other gastrointestinal symptoms. She had no sick contacts or potential exposures for foodborne illnesses. No other joints were affected. There were no systemic, mucocutaneous, pulmonary, uro-gynaecological, and visual features; no jaw claudication. There were no preceding infective or prodromal symptoms.

On examination, there was no effusion, erythema or warmth of the affected joints. Of note, examination revealed complete shoulder range of motion, said to have improved compared to previous days and attributed to slightly improving pain. There was, however, ongoing restricted range of motion and difficulty weightbearing on the knees.

Complete blood count and complete metabolic panel undertaken on the same day were within normal limits. There was elevated C-reactive protein at 93.8 mg/L and erythrocyte sedimentation rate of 30 mm/h. Serology was unremarkable, including for rheumatoid factor, antineutrophilic cytoplasmic antibodies and extractable nuclear antigen antibodies.

The patient was reviewed two days later with improving symptoms, aided by a walking stick and minimal assistance, but without wheelchair use. She was again reviewed on 27 May with complete resolution of her signs and symptoms, with corresponding C-reactive protein of 5.8 mg/L on 26 May 2021. Her erythrocyte sedimentation rate remained at 30 mm/h and her lactate dehydrogenase rose from 176 to 393 U/L.

The patient was initially hesitant about receiving further doses of ChAdOx1 nCov-19 vaccine, but was counselled about the risks and benefits of receiving her second dose, as well as the decreased reactogenicity following second dose compared to after the first dose [1]. She ultimately presented for her second dose of ChAdOx1 nCov-19 vaccine on 13 August 2021, and did not develop any significant adverse reactions when followed up on 8 September 2021.

## 3. Discussion

Here we presented a case of an elderly recipient of the ChAdOx1 nCov-19 vaccine with good baseline mobility, no pre-existing rheumatological diagnosis, and no epidemiological exposures to COVID-19, who presented with new onset polyarthralgia and severe functional impairment following her first dose of the vaccination. The symptomology and progression within a plausible time window, in the absence of any other inciting events, and in the setting of known similar presentations (as discussed further below), led us to assess this case of acute polyarthralgia as consistent with an association to the patient’s immunization with the ChAdO1 nCov-19 vaccine [7]. While these were self-limiting in this patient, the episode represents a significant traumatic experience for the patient.

While arthralgia had been a common adverse reaction following the ChAdOx1 nCov-19 vaccine, the pattern of polyarthralgia present in this patient and its delayed onset of 16 days following vaccination make this case distinctive. The subsequent severe functional impairment in a 79-year-old patient is also a departure from available data and reports. Data from phase 2/3 trial of the vaccine [1] shows only 7/49 participants in the ≥70 age group reporting joint pain, and none of these participants experienced any functional impairment as a result of their joint pain. These reports of joint pain as a solicited adverse reaction were recorded by participants within the first seven days post-vaccination, making it a limitation in the assessment of delayed reactions post-vaccination. In our patient, the onset of fatigue within 12 h post-vaccination, its persistence to the onset of polyarthralgia and diarrhea, and its temporally related resolution increase the possibility of this constellation of symptoms to be vaccine related.

At initial presentation, our patient’s age and female gender, arthralgia involving the neck, bilateral shoulders, right hip and both knees, elevated acute phase reactants, aches worse in the morning, lasting longer than 45 min, and normal serology, led to the consideration of polymyalgia rheumatica, and for giant cell arteritis to be ruled out. Importantly, Hyun et al. recently reported five patients from a tertiary hospital in Korea who developed polyarthralgia and myalgia mimicking rheumatologic diseases, including polymyalgia rheumatica and rheumatoid arthritis [8]. A limitation of this case report is the lack of stool sample for gastroenteric pathogens to be definitively ruled out as an alternate cause of presentation, although the absence of common features including fever, abdominal pain, blood in stools, nausea and vomiting and other gastrointestinal symptoms decrease the likelihood of this possibility.

Of five patients reported by Hyun et al. [8], four were elderly (aged ≥ 65 years), all were female, had no pre-existing rheumatological conditions and had elevated erythrocyte sedimentation rate, C-reactive protein and lactate dehydrogenase levels. Symptoms persisted between 10 and 47 days, in contrast to Ramasamy et al.’s findings of rapid resolution of symptoms [1]. A 25-year-old patient experienced similar symptoms following her second dose of ChAdOx1 nCov-19 vaccine, which could be seen as a positive re-challenge observation [7,8]. On the other hand, the lack of symptom recurrence in our patient, as well as in the four elderly patients reported by Hyun et al., could be interpreted as a negative re-challenge observation [7,8]. These findings are, however, consistent with Ramasamy et al.’s findings of decreased reactogenicity with increasing age, and fewer adverse reactions following the second dose than after the first dose [1].

New onset rheumatological conditions and flares of existing, well-controlled disease after SARS-CoV-2 infection have been established [9,10,11,12]. Reports of these following vaccination against SARS-CoV-2 exist to a lesser extent [13,14]. Viral arthralgias as a distinct clinical entity following SARS-CoV-2 infection is only beginning to be recognized [15]. It is possible that this case report represents a similar entity which occurs following vaccination against SARS-CoV-2; although the underlying mechanism(s) behind such reactions remains to be investigated. In the case of the ChAdOx1 nCov-19 vaccine, activation of proinflammatory cytokines had been speculated as the underlying mechanism of systemic inflammatory arthralgia [8], whereas genetic predisposition and local deposition of immune complexes had been previously suspected in arthralgia following vaccination against HBV and rubella, respectively [16,17].

The potential of SARS-CoV-2 to cause severe disease, lasting morbidity and mortality especially among older adults means the benefits of vaccination continue to outweigh the risk of any polyarthralgia or myalgia, especially if self-limiting.

Our case report confirms the observations of Hyun et al. [8] outside the Korean setting. To our best knowledge, this is also the first report of severe, albeit self-limiting, functional impairment following ChAdOx1 nCov-19 vaccine in an elderly patient. There remains a possible association between the ChAdOx1 nCov-19 vaccine and polyarthralgia, with diarrhea, although a gastroenteric pathogen as etiology of this presentation was not definitively ruled out. The information presented in this case report, along with future dissemination of clinical information from surveillance of adverse events, is valuable in aiding patient discussion when such presentations occur, and also in aiding with the clinical decision to weigh up further investigation or empirical treatment, against the risks and benefits of reassurance and close monitoring.

## Data Availability

The data of this study is available on request from the corresponding author.
